# Effect of Coronavirus Disease 2019 in Pulmonary Circulation. The Particular Scenario of Precapillary Pulmonary Hypertension

**DOI:** 10.3390/diagnostics10080548

**Published:** 2020-07-31

**Authors:** Jorge Nuche, Teresa Segura de la Cal, Carmen Jiménez López Guarch, Francisco López-Medrano, Carmen Pérez-Olivares Delgado, Fernando Arribas Ynsaurriaga, Juan F. Delgado, Borja Ibáñez, Eduardo Oliver, Pilar Escribano Subías

**Affiliations:** 1 Centro de Investigaciones Biomédicas En Red de enfermedades CardioVasculares (CIBERCV), 28029 Madrid, Spain; jorge-nuche@hotmail.com (J.N.); cjlguarch@gmail.com (C.J.L.G.); fernando.arribas@salud.madrid.org (F.A.Y.); juan.delgado@salud.madrid.org (J.F.D.); bibanez@cnic.es (B.I.); 2 Servicio de Cardiología, Hospital Universitario 12 de Octubre, Instituto de Investigación Sanitaria Hospital, 12 de Octubre (imas12), 28041 Madrid, Spain; teresaseguradelacal@gmail.com (T.S.d.l.C.); carmenperezolivaresd@gmail.com (C.P.-O.D.); 3 Centro Nacional de Investigaciones Cardiovasculares, 28029 Madrid, Spain; 4 Facultad de Medicina, Universidad Complutense de Madrid, 28040 Madrid, Spain; flmedrano@salud.madrid.org; 5 Department of Infectious Diseases, Hospital Universitario 12 de Octubre, Instituto de Investigacioón Sanitaria Hospital 12 de Octubre (imas12), 28041 Madrid, Spain; 6 IIS-Fundación Jiménez Díaz, 28040 Madrid, Spain

**Keywords:** pulmonary hypertension, COVID-19, pulmonary circulation, endothelial dysfunction

## Abstract

The Coronavirus Disease of 2019 (COVID-19) has supposed a global health emergency affecting millions of people, with particular severity in the elderly and patients with previous comorbidities, especially those with cardiovascular disease. Patients with pulmonary arterial hypertension (PAH) and chronic thromboembolic pulmonary hypertension (CTEPH) could represent an especially vulnerable population because of the high mortality rates reported for respiratory infections. However, the number of COVID-19 cases reported among PAH and CTEPH patients is surprisingly low. Furthermore, the clinical picture that has been described in these patients is far from the severity that experts would expect. Endothelial dysfunction is a common feature between patients with PAH/CTEPH and COVID-19, leading to ventilation/perfusion mismatch, vasoconstriction, thrombosis and inflammation. In this picture, the angiotensin-converting enzyme 2 plays an essential role, being directly involved in the pathophysiology of both clinical entities. Some of these common characteristics could explain the good adaptation of PAH and CTEPH patients to COVID-19, who could also have obtained a benefit from the disease’s specific treatments (anticoagulant and pulmonary vasodilators), probably due to its protective effect on the endothelium. Additionally, these common features could also lead to PAH/CTEPH as a potential sequelae of COVID-19. Throughout this comprehensive review, we describe the similarities and differences between both conditions and the possible pathophysiological and therapeutic-based mechanisms leading to the low incidence and severity of COVID-19 reported in PAH/CTEPH patients to date. Nevertheless, international registries should look carefully into this population for better understanding and management.

## 1. Introduction

The coronavirus disease of 2019 (COVID-19) is an infectious disease that emerged in the Chinese city of Wuhan in December 2019 [1] and rapidly extended worldwide, leading to its declaration as a pandemic disease by the World Health Organization on 11 March 2020 [2]. By 30 June 2020, the rapid spread of the virus has caused more than 10,000,000 cases and more than 500,000 deaths all over the world (source: https://www.worldometers.info/coronavirus/). 

COVID-19 is caused by the severe acute respiratory coronavirus 2 (SARS-CoV2), an RNA virus of the family Coronaviridae. Common symptoms include fever, cough, sore throat and dyspnea [1,3], while the most severe cases develop pneumonia and acute respiratory distress syndrome (ARSD), requiring admission to the intensive care unit in up to 20% of hospitalized patients [4]. Reported data suggest an overall mortality rate ranging from 0.3 per 1000 cases in patients aged under 18 to 305 per 1000 cases in elderly people (>85-year-old) [4]. 

Precapillary pulmonary hypertension (PH) includes a variety of diseases leading to increased pulmonary artery pressure (≥25 mmHg) in the presence of normal pulmonary capillary wedge pressure (<15 mmHg) and a pulmonary vascular resistance >3 Wood units at rest [5]. Among them, pulmonary arterial hypertension (PAH) and chronic thromboembolic pulmonary hypertension (CTEPH) constitute a particular group due to its relation to a poor quality of life and shortened survival [6]:

- PAH is a rare, noncurable disease characterized by an aberrant pulmonary vascular remodeling that may be either idiopathic or related to different clinical conditions [6]. PAH-related histological changes include endothelial damage and dysfunction, vasoconstriction, vascular cell proliferation leading to vascular obliteration and wall thickening and, eventually, to the formation of plexiform lesions [7]. Furthermore, there is a marked component of perivascular inflammation and microthrombosis [8].
- CTEPH constitutes a different group of precapillary PH secondary to the obstruction of the pulmonary arteries by organized thrombus after a pulmonary embolism. These pulmonary vascular changes induce small-vessel vasculopathy consisting on altered vascular remodeling initiated or potentiated by a combination of defective angiogenesis, impaired fibrinolysis and endothelial dysfunction [9].

The resultant increased pulmonary arterial pressures in PAH and CTEPH contribute to a further increase of the right ventricular afterload, thus leading to right ventricular dysfunction and reducing survival among affected patients, being heart failure the most common cause of death [10]. Hospitalizations for cardiovascular and non-cardiovascular diseases are common in these groups of patients and carry a high mortality risk, being especially high in patients admitted to the intensive care unit [11,12]. 

Patients with previous cardiovascular risk factors or a cardiovascular disease seem to be at a higher risk of developing severe forms of SARS-CoV-2, with higher rates of mortality described in this population [1,13,14]. Moreover, the shortage of equipment needed to care for critically ill patients in some areas has led to difficult decisions, and patient’s short-term likelihood of surviving the acute medical episode has remained the rationale to rationing. As physicians, bearing this reality in mind, it seemed straightforward to assume that PAH and CTEPH patients were at a higher risk of developing severe forms of the disease and that their chances of recovering from such an insult were scarce. 

Noteworthy, some case-series have repeatedly presented the unexpected favorable outcome of COVID-19 infection in this population, suggesting that these patients could be somehow protected against severe forms of the disease [15,16,17]. However, whether this protection is due to receiving therapy, to their special awareness about their particular risk factor or to any other physiological condition related to the disease is still a remaining question. 

**Figure 1:**
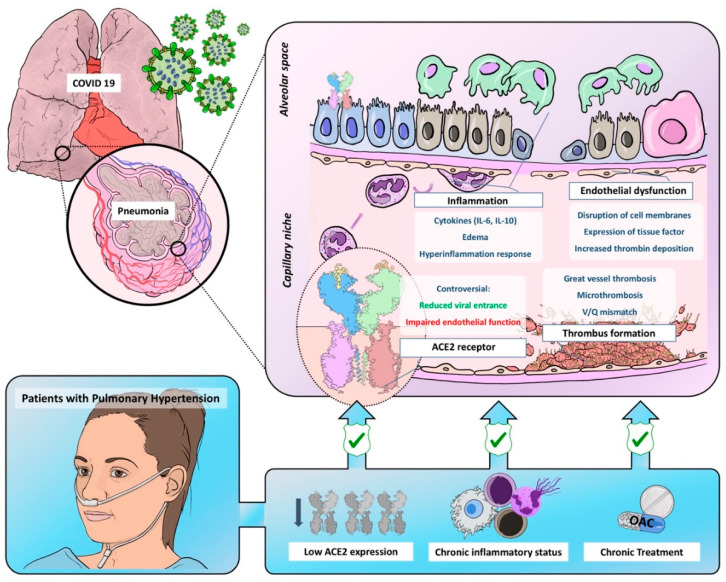
Graphic representation of common pathobiological characteristics between Coronavirus Disease of 2019 and pulmonary hypertension and the possible mechanisms leading to a reduced virulence and severity in this population. ACE2 = angiotensin-converter enzyme 2; OAC = oral anticoagulant.

With this comprehensive review, we aim to describe the effects of SARS-COV-2 in the pulmonary circulation and the possible differential features in patients exhibiting pulmonary microvasculopathy, such as PAH and CTEPH patients, since we believe it will help to further understand COVID-19 and its pulmonary consequences ((Figure 1).

## 2. Endothelial Dysfunction and COVID-19

Endothelium has paracrine, endocrine and autocrine functions necessary for the homeostasis of the vessels in all vascular territories. Consequent to the endothelial barrier damage, a dysfunctional endothelium will lead to widespread vasoconstriction, disruption of the vascular permeability, leakage and cytokine release, which, eventually, will drive to inflammation and a procoagulant state [18].

Autopsy studies of the lungs of patients dying from COVID-19 describe diffuse alveolar damage and perivascular lymphocytic infiltrate. However, the distinctive tissue findings in COVID-19 lungs are the severe endothelial damage and the disruption of cell membranes associated with the identification of the intracellular virus but, also, related to exacerbated inflammatory cell infiltrations in late stages of the disease [18,19,20,21]. In addition, pulmonary vessels show extensive thrombosis and microangiopathy, being even more severe in COVID-19 patients than in influenza H1N1 patients [19]. This procoagulant state is not limited to the development of great vessel thrombosis but to micro-vessel thrombosis, which may contribute to the development of pulmonary ventilation/perfusion (V/Q) mismatch with increased death space. 

Thus, there is growing evidence suggesting that COVID-19 pathobiology extends beyond the inflammatory response to the viral infection, with a clear implication of endothelial damage leading to coagulopathy, vasoconstriction and inflammation, paramount for the pathogenesis of severe lung damage and COVID-19 severity. However, up-to-date further research is needed for better understanding the role of the endothelium and the influence of the endothelial barrier integrity in the development and progression of COVID-19 patients. 

## 3. Thrombosis and COVID-19

Thrombotic events are common in COVID-19 patients, with high rates of stroke, myocardial infarction, venous thrombosis and pulmonary embolisms described in the literature [22,23]. In addition, abnormal coagulation parameters have also been described, with a poorer prognosis for those patients with increased D-dimers or prothrombin time [24] and coagulation parameters, meeting the criteria for disseminated intravascular coagulation in up to 71.4% patients dying from COVID-19 [25]. Furthermore, different observational studies suggest an improved survival associated with the administration of prophylactic and therapeutic doses of anticoagulant treatments in selected patients [26,27].

Based on these clinical observations, local protocols and medical societies recommend D-dimer, prothrombin time, platelet count and fibrinogen level evaluations in all patients to assess the short-term prognosis, recommending admission for those with markedly altered hemostatic parameters [28]. At the moment, a prophylactic dose of low molecular weight heparin is recommended to all patients admitted to hospital independently of the presence of altered coagulation parameters [28]. Furthermore, several randomized clinical trials evaluating the effectiveness of anticoagulation in COVID-19 are currently ongoing (Table 1). 

Thus, vascular thrombosis, both at a microscopic and macroscopic level, is one of the distinctive features of COVID-19, having been reported in up to 24% of cases. This contrasts with the usual incidence in other viral infections, such as 6% for the H1N1 influenza A infection [29].

With regards to the macroscopic thrombosis burden in the pulmonary arterial bed, a pulmonary embolism pattern in COVID-19 patients is characterized by a lower thrombotic load and a distribution mainly through segmental arteries [30]. This probably translates that the reason for the pulmonary arterial thrombosis seen in these patients lies mainly in the endothelial dysfunction and the inflammation accompanying COVID-19, rather than a thrombotic migration from the venous system [31]. Noteworthy, right ventricular function appears to be less impaired in the setting of COVID-19 pulmonary embolism than it usually is in common pulmonary embolism, which probably translates a lower increase in pulmonary pressures [31]. 

This thrombogenic environment appears to be secondary to the endothelial dysfunction, resulting in an increased thrombin deposition and impaired fibrinolytic pathways, but it is also indicative of the hypercoagulability status characteristic of patients suffering infectious and inflammatory disorders [32]. 

## 4. Inflammatory Response in COVID-19 

Severe COVID-19 is characterized by a disproportional inflammatory response to an initial acute lung insult, which aggravates the lung injury and leads to a severe hypoxemic respiratory failure and, ultimately, to death [33]. Thus, in the severe cases, the initial adaptive immune response necessary to eliminate the virus is followed by a severe and persistent inflammatory reaction, which appears to govern the clinical picture at this point, leading to further lung destruction and, therefore, ARDS. This exaggerated inflammation is mediated by several cytokines (IL-6, IL10, TNF-α, INF-γ), as well as mononuclear cells and neutrophils [33,34]. IL-6 is the central element of this cytokine storm by stimulating the expression of other cytokines and promoting edema [35]. The level of cytokines is higher in those patients who require admission to intensive care units in whom their lymphocyte count is lower. This finding suggests that the low absolute lymphocyte count observed in COVID-19 patients could be a consequence of the high serum cytokines concentrations that negatively regulate T-cell survival and proliferation [36]. Furthermore, T cells in COVID-19 express high levels of programmed cell death-1, an indicator of T-cell exhaustion [36]. Thus, it seems that the severe clinical picture described in COVID-19 patients is mediated by this inflammatory response responsible for both lung injury and impaired cellular immunity, which hampers the fight against the viral infection [36]. 

This immune response to the SARS-Cov-2 infection is more severe than that produced in other viral infections [37]. Older patients and those with previous comorbidities, which may present a chronic subclinical inflammatory status, could be at a higher risk of developing this cytokine storm. This could explain, at least in part, the worse prognosis described in these groups [14,37,38]. 

On histology, the lungs of COVID-19 patients present diffuse alveolar damage with “necrosis of alveolar lining cells, pneumocyte type 2 hyperplasia and linear intra-alveolar fibrin deposition” [19]. The inflammatory infiltrate described in COVID-19 lungs differs from that described in H1N1 influenza lungs, with a predominant infiltration of CD4-positive T cells and a lower number of CD8-positive T cells and neutrophils in COVID-19 lungs [19]. Furthermore, there is also an extensive macrophagic infiltration contributing to the alveolar damage [39] 

These inflammatory and the above-described procoagulant statuses are not isolated phenomena. The mechanisms leading to the activation of a coagulation cascade in COVID-19 are not well-defined but seem to be related to the inflammatory response also responsible for the endothelial damage and include the releasing of procoagulant factors [33,40]. COVID-19 pneumonia has some clinical, analytic and histological similarities with macrophage activation syndrome (MAS), including the cytokine storm and high ferritin levels [39,41]. In fact, this is the rationale for the use of anti-cytokine therapies and corticosteroids in severe COVID-19 patients [41,42,43,44]. This MAS-like inflammatory response triggers the expression of the active tissue factor, which leads to a massive coagulation cascade activation, with the ulterior development of thrombotic events and widespread pulmonary microangiopathy [39]. These endothelial damages and coagulation activations are enhanced by a local hypoxia constituting a deleterious vicious circle [39,45]. 

Furthermore, the detection of serum marker-specific neutrophil extracellular traps (NETs) in COVID-19 patients suggests a pivotal role of these structures in the marked inflammatory and prothrombotic responses. NETs are extracellular webs of chromatin, microbicidal proteins and oxidative agents released by neutrophils to contain infections. The uncontrolled release of NETs leads to a procoagulant status and widespread inflammation [21]. Thus, an increased neutrophil-to-lymphocyte ratio is related to a severe COVID-19 course due to a disproportionate innate immune response leading to the release of cytokines and inflammatory infiltrates with tissue necrosis [46].

Thus, it seems that severe ARDS and widespread thrombosis are complementary phenomena leading to the severe clinical course described in COVID-19 patients [19].

## 5. Pulmonary Vasculature Dysfunction 

COVID-19 has a deleterious effect in pulmonary circulation that is driven by the above-mentioned widespread endothelial damage and micro-thrombosis leading to ventilation/perfusion (V/Q) mismatch but, also, by changes in the vascular tone that could aggravate hypoxemia [47,48,49]. 

The classic behavior of ARDS lungs is characterized by the presence of non-cardiogenic pulmonary edema (infiltration of the alveolar space by inflammatory infiltrates and fibrins), a hypoxemia secondary to intrapulmonary shunts and low compliance [48]. In these cases, the preferred strategy includes “protective” ventilation with low tidal volume and high positive end-expiratory pressure to protect the lungs. Furthermore, these patients usually respond properly to recruiting maneuvers and prone positions [48].

In COVID-19 patients, two different ventilatory patterns have been described: Some patients present normal or nearly normal compliance with severe refractory hypoxemia without a response to recruiting maneuvers or high positive end-expiratory pressure (type L), and the other group of patients present an ARDS-like lung (type H) [47,48]. The impaired regulation of hypoxic vasoconstriction, with a deviation of blood flow to poorly ventilated areas leading to a intrapulmonary shunt effect, has been proposed as the cause of the finding characteristics of type L lungs in which responses to a prone position might be driven by gravitational changes of the blood flow [47]. Ultimately, the damage inflicted to the lungs both by the respiratory effort of the patients and that associated to mechanic ventilation leads to the appearance of a “classic” ARDS pattern [48]. 

Hypoxic pulmonary vasoconstriction is a homeostatic response in which the vasoconstriction of the pulmonary bed in those poorly ventilated lung segments leads to a deviation of the blood flow to well-ventilated segments, improving V/Q matching [50]. Some authors suggest that COVID-19 patients may have an exaggerated pulmonary vasoconstriction mimicking high-altitude pulmonary edema and propose the use of acetazolamide, calcium channel blockers and phosphodiesterase-5 inhibitors for the treatment of COVID-19 pneumonia [49,51]. However, high-altitude pulmonary edema lacks neutrophil infiltrations or a cytokine storm and is a consequence of an abnormally high hydrostatic pressure [52], and the opposite mechanism has also been proposed, in which a hampered hypoxic vasoconstriction is responsible of the intrapulmonary shunt leading to hypoxemia [47,48,50]. This weak vasoconstrictor response might be related to an impaired carotid body function as a consequence of changes in the expression of mitochondrial proteins related to oxygen-sensing mechanisms, and these authors propose the use of inhibitors of endogenous vasodilator pathways to reduce intrapulmonary shunts [50]. However, the marked differences between both conditions prevent us from recommending the routine administration of specific pulmonary vasodilators [53].

The role of pulmonary circulation damage in COVID-19 severity is therefore controversial and complex, with a component of both V/Q mismatch and an intrapulmonary shunt that might be even simultaneous. A hemodynamic evaluation of COVID-19 patients with pulmonary artery catheters would be useful to improve our knowledge of the disease. 

## 6. Angiotensin Converter Enzyme 2: An Actor in a Leading Role

The role of angiotensin converter enzyme 2 (ACE2) in COVID-19 has been widely discussed, as this transmembrane receptor is used by SARS-COV-2 to pass cells’ membranes [54,55], and its expression is positively related with infectivity [56]. In fact, some concerns have arisen about the safety of angiotensin-converter enzyme inhibitors and angiotensin-2 type-1 receptor blockers, since they are known to upregulate the expression of membrane ACE2 [57,58] and, therefore, could facilitate viral entrances into the cells. However, observational and randomized studies have not shown any deleterious effects related to these drugs, and some other studies even suggest a beneficial effect of its administration [59,60,61,62]. 

Angiotensin-converter enzyme (ACE) and ACE2 have counterbalancing effects and are implicated in the regulation of pulmonary and systemic vascular beds [59,63]. ACE cleavages angiotensin to angiotensin-II, which, through angiotensin-II receptor type 1 pathway activation, leads to vasoconstrictor proliferative and prothrombotic responses. On the contrary, ACE2 cleavages angiotensin-II to the heptapeptide angiotensin-(1-7), which counter-regulates the action of angiotensin-II with vasodilator antithrombotic and antiproliferative effects [59,63]. Thus, reducing the ACE2 role in the severity of COVID-19 due to its implication in viral infectivity could be too simplistic, since this receptor has demonstrated to be involved in the pathogenesis of the different above-described mechanisms leading to lung and cardiovascular damage. 

ACE2 has an endothelial protective effect by counter-regulating the effect of the ACE angiotensin II. In preclinical studies, an overexpression of ACE2 has shown to reduce atherosclerosis by protecting the endothelial function and, also, by inhibiting the inflammatory response through a reduced expression of MCP-1, VCAM-1 and E-selectin induced by angiotensin II [64]. This endothelial protective effect is also responsible for an antithrombotic effect. In this sense, the ACE2 expression has shown to be inversely correlated with a thrombotic burden in patients with pulmonary embolism [65]. Moreover, in animal models of thrombosis, a reduced ACE2 activity is related to a higher thrombus volume. This prothrombotic effect is enhanced by the administration of ACE2 inhibitors and reversed with the administration of ACE2 activators [66].

Regarding the inflammatory response, ACE2 participates in the regulation of innate immunity, and its overexpression is able to reduce cytokine releases [39,67]. In an experimental setting, a genetic deletion of ACE2 in a mice model of pulmonary inflammation was related to bigger lung damage after acid inhalation than in wild-type mice [55]. The SARS-CoV spike protein binding to ACE2 downregulates the ACE2 expression and induces lung injury through angiotensin II receptor type 1 activation by angiotensin II [55]. The dynamic change of ACE2 expression on pneumocytes’ surfaces is essential to control neutrophilic inflammation during bacterial infections. Thus, at an initial stage of the infection, a reduced ACE2 expression facilitates the development of an inflammatory response to neutralize the aggression. In later stages, a recovered ACE2 expression would help to control the inflammatory infiltrate, avoiding disproportionate injury [46]. 

Thus, the SARS-Cov-2 spike protein binds to ACE2 to enter the cell and reduces the surface ACE2 expression both through its internalization and through its binding to endogenous ACE2, which results in ACE2 downregulation [55]. This reduced ACE2 expression leads to a diffuse endothelial dysfunction, leading to severe lung damage with an inflammatory infiltrate and edema, widespread thrombosis and changes in the vascular homeostasis [68].

Whether a low ACE2 expression could be beneficial (due to a reduced viral entrance) or harmful (due to an impaired endothelial function) in the course of COVID-19 has generated some controversy. Although there are no available data suggesting a deleterious effect of drugs producing ACE2 overexpression, solid evidence to solve the dilemma is lacking. However, the absence of an association between antihypertensive drugs blocking the renin-angiotensin axis and adverse outcomes must be highlighted to avoid adverse events related to a nonjustified withdrawal in chronic users. 

## 7. Pulmonary Hypertension: A Paradigm of Endothelial Dysfunction

We have described how SARS-Cov-2 has an affinity for endothelial cells affecting not only to pulmonary circulation but to other vascular territories (heart, gut, brain and kidney). This becomes even more evident in patients with previous endothelial dysfunctions, such as hypertension or diabetes mellitus [18].

Endothelial dysfunction is one of the most relevant hallmarks and a critical contributor determining the onset and progression of PH. Pulmonary vascular endothelium controls the blood barrier integrity, the vascular tone by producing vasodilator and vasoconstrictor molecules and, also, optimizes the gas exchange in different conditions, being a main player in maintaining the vasomotor balance and vascular-tissue homeostasis. In addition, the integrity of the endothelial barrier is essential for controlling an inflammatory and thrombosis-free surface. Therefore, an alteration of the endothelium leads to a pro-edematous, pro-vasoconstriction, prothrombotic and proinflammatory phenotype, which eventually alters the cell metabolism and oxidative stress, leading to a pro-proliferative and antiapoptotic phenotype all responsible in the last instance of the PH phenotype [31,69,70]. 

Pulmonary vascular remodeling in PAH and CTEPH is not only caused by the proliferation of different cells in the arterial wall but by the loss of precapillary arteries and by a severe chronic perivascular inflammatory infiltrate [8]. These conditions constitute a paradigm of endothelial dysfunction with a propensity to vasoconstriction; thrombosis; the production of reactive oxygen species; the expression of adhesion molecules (E-selectin, ICAM-1 and VCAM-1) and a massive release of cytokines and growth factors [8]. Thus, a devastating effect of COVID-19 would be expectable in these patients.

**Figure 2:**
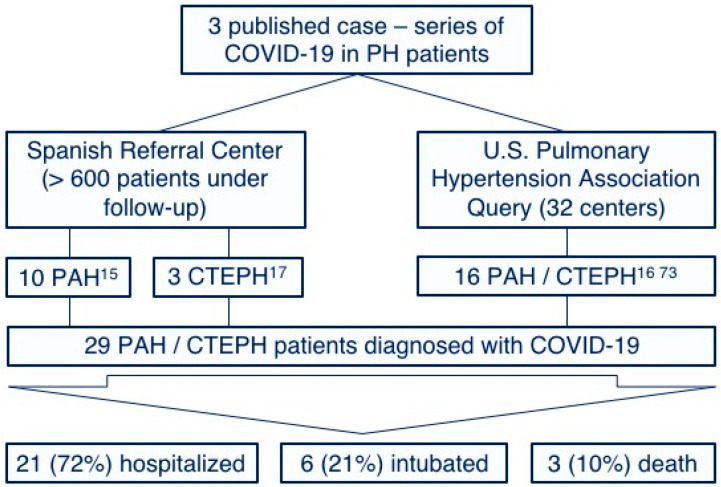
Reported cases and outcomes of the coronavirus disease of 2019 among pulmonary arterial hypertension and chronic thromboembolic pulmonary hypertension patients. PH: Pulmonary hypertension, PAH: Pulmonary arterial hypertension and CTEPH: Chronic thromboembolic pulmonary hypertension.

Few cases of COVID-19 in PAH and CTEPH patients have been reported. This observation may be explained by the low prevalence of these diseases and by the increased awareness of chronic patients with a special emphasis on social-distancing measure compliances [71,72]. However, we have recently published our surprisingly positive experience with COVID-19 in our PAH [15] and CPTEH [17] populations without any of them requiring intensive care and any deaths reported. Furthermore, a similar benign course has been observed in other centers [16,73], with no published reports suggesting particularly adverse outcomes [74] (Figure 2). Although the proportion of patients requiring intubation is similar to that described for the global COVID-19 population [4], it must be highlighted that these case-series represent a very biased population constituted only by those symptomatic patients who required hospitalization. We hypothesize that a high proportion of PAH and CTEPH patients infected by SARS-Cov-2 might have developed a mild or even an asymptomatic course and were not tested for SARS-Cov-2. Thus, the low prevalence of COVID-19 in PH patients might also be explained by this low proportion of patients with severe symptoms requiring medical attention (and, subsequently, being tested for SARS-Cov-2). The Spanish pulmonary hypertension registry is currently collecting information about all PAH and CTEPH patients diagnosed with COVID-19, aiming to improve our knowledge on the prognostic implications of the disease in this population. 

The chronic pathological findings in PAH and CTEPH somehow remind of those found in COVID-19 specimens. In fact, some experimental treatments tested for PAH such as recombinant ACE2, synthetic vasoactive intestinal peptide or IL-6 antagonists are already being tested in clinical trials for COVID-19 pneumonia, which support the hypothesis of pathophysiological similarities between both conditions [75] (Table 1). This makes this population extremely vulnerable to acute conditions, especially to those causing severe respiratory failure [11]. In fact, infectious conditions are a common cause of decompensated right heart failure and death in these patients [74]. 

Although our observations of a benign course in PAH and CTEPH patients requires caution, we described some pathophysiological characteristics that could explain this benign course [15,17]. 

### 7.1. Chronic Inflammatory Status 

A growing body of evidence has demonstrated the accumulation of a variety of inflammatory and immune cell infiltrations in PH lungs in and around the wall of remodeled pulmonary resistance vessels and in the vicinity of plexiform lesions, regardless of the cause of PH [76]. Thus, patients with PAH have a different immune cellular landscape, which suggests a shift toward the adaptive immune system. Furthermore, high cytokine levels also take part in the pathobiology of pulmonary vascular remodeling [8,77], and this molecular pathway constitutes a promising target for future treatments aiming to reduce this adverse remodeling [77].

Two different hypotheses could explain the role of this chronic inflammatory status in the reduction of COVID-19 severity. First, the presence of this extra-immune perivascular and perialveolar system layer (“tertiary lymphoid tissue”), which could limit the viral infection and expansion [15]. However, this protective effect has not been reported for other bacterial or viral infections in PAH and CTEPH patients, and they are considered a high-risk population, with an indication for vaccination against Streptococcus pneumoniae and for annual vaccinations against influenza [5]. Second, this baseline inflammatory status could be responsible of the “exhaustion” of the innate immune response limiting the “step-up” necessary to unleash the cytokine storm [16]. 

### 7.2. Low ACE2 Expression in Pulmonary Hypertension: Yin or Yang?

In PAH patients, there is a reduced expression of surface and plasmatic ACE2 [78]. This contributes to the endothelial dysfunction characteristic of these patients, which ultimately leads to a proinflammatory, prothrombotic and hyperproliferative status responsible for pulmonary vascular remodeling [78]. For this reason, recombinant ACE2 has been proposed as a treatment for PAH, with the aim to reduce pulmonary vascular remodeling and microvasculature thrombosis, leading to V/Q disturbances [79]. 

The reduced ACE2 expression in CTEPH has not been specifically evaluated. However, given the well-described relation of ACE2 under-expression and thrombotic events, low ACE2 levels among CTEPH patients is also expected [63]. 

We have previously hypothesized that this reduced ACE2 expression could confer a protective status for this population, due to a reduced viral capacity to infect pneumocytes [15,17]. However, since ACE2 has also a protective effect on endothelial cells, the previously described dilemma takes special interest in this matter [59]. 

Thus, although low ACE2 levels might theoretically make PH patients more vulnerable to severe forms of COVID-19, the hampered viral entrance in these patients could act as an initial barrier that would stop the development of a massive inflammatory response.

### 7.3. Hypothetical Protective Effect of Chronic Treatment in PH Patients 

The pathophysiological characteristics of COVID-19 patients described above might justify the administration of specific treatments for PAH and CTEPH. Although endothelial dysfunction is a key factor responsible for the altered pulmonary perfusion in these patients, the chronic treatment they receive may somehow protect them against the additional acute damage due to the SARS-CoV-2 infection. 

#### 7.3.1. Anticoagulation 

As described above, a prothrombotic status has been recognized in COVID-19 that can be at least partially explained by the already-mentioned induced endothelial dysfunction, the disproportionate inflammatory response but, also, to the reduced ACE2 expression induced by the SARS-Cov-2 infection [40]. Thus, there is broad consensus about the potential beneficial effects of prophylactic heparin doses in COVID-19 pneumonia. Some studies even suggest an additional benefit of anticoagulant doses to further offset this pathogenic mechanism [26]. Besides its anticoagulant role, additional anti-inflammatory effects have been attributed to heparin [80]. CTEPH patients routinely receive chronic systemic anticoagulation (usually, vitamin K antagonists) to avoid repeated embolic events [5] and, although not as a primary indication [5], many PAH patients are also anticoagulated due to prothrombotic comorbidities. This fact may confer a protective status against the widespread thrombosis seen in COVID-19 and could partially explain the low number of severe clinical pictures in PH patients, especially in CTEPH. 

#### 7.3.2. Specific PH Therapy 

As described above, two opposite hypotheses have been described in COVID-19-related ARDS: An abnormal hypoxic vasoconstriction contributing to the deviation of blood flow precisely to those alveolar units poorly ventilated [47,48] and a high-altitude pulmonary edema physiology [49]. Although the last hypothesis would clearly justify the use of specific pulmonary vasodilators, it has been openly rejected by different groups due to clear pathophysiological differences among both syndromes and to the lack of data suggesting a severe pulmonary artery pressure increase as the cause of refractory hypoxemia in COVID-19 [53].

The potential therapeutic role of specific PH therapies in the setting of ARDS has been widely discussed. ARDS suffer severe hypoxemia related to intrapulmonary right-to-left shunts, and specific pulmonary vasodilators are commonly used to shift the blood flow to properly ventilated areas [81]. Furthermore, these treatments have also an anti-inflammatory effect and a beneficial effect on endothelial homeostasis [82]. 

In PH patients, phosphodiesterase-5 inhibitors improve the gas exchange by dilating pulmonary bed vessels of properly ventilated areas [83]. Based on this pharmacological property, these drugs have been proposed as a potential treatment for ARDS, although no solid evidence favoring its systematic use has been obtained, as it has shown to increase the shunt fraction [84]. However, sildenafil has also proven an anti-inflammatory and endothelium-protective effect that may confer a benefit for COVID-19 patients [85]. There is currently ongoing one single-arm clinical trial to assess its effectivity.

Endothelin-1, by binding to endothelin type B receptor, increases the vascular tone but, also, participates in the chronic inflammatory status described in PH patients by inducing cytokine, growth factors, collagen and aldosterone production [8,86]. Furthermore, it has also shown to have an important role in the development of ARDS via the angiotensin type A receptor. In this regard, in preclinical studies, bosentan, an endothelin receptor antagonist, has shown to be effective for the treatment of ARDS due to its anti-inflammatory effects, which includes a reduced neutrophil infiltration and plasma extravasation [86]. Furthermore, its use in humans for this purpose has also been reported [87]. The use of endothelin receptor antagonists for COVID-19 has already been proposed [88], and the use of ambrisentan in the treatment of COVID-19 is being tested in a randomized clinical trial.

The use of inhaled nitric oxide in ARDS is widely extended, since it has been shown to improve the oxygenation in intubated patients, as well as the radiographic pattern [89]. Although the use of inhaled nitric oxide for ARDS has shown controversial results, the most consistent benefit has been proven precisely in coronavirus-related ARDS [90]. This fact may be explained by the nitric oxide capacity to reduce viral replications [91,92]. However, nitric oxide inhalation does not reduce mortality or ventilator-free days, and it is related to increased renal impairment [93]. The potential benefit of inhaled nitric oxide is also being evaluated in clinical trials. 

The use of inhaled prostacyclins has also been proposed for refractory ARDS based on the same pathophysiological rationale of other specific pulmonary vasodilators (improved perfusions of well-ventilated alveoli). However, as for the above-mentioned treatment, robust evidence justifying its routine use in ARDS is lacking [81].Despite controversial hypotheses, we do believe that specific PH treatments could have contributed to a less severe course of COVID-19 in our cohort of patients by two mechanisms: A protective effect over the endothelium and its contribution to the maintenance of a homogeneous vascular tone, avoiding the increase of the intrapulmonary shunt described in COVID-19. 

The above-described hypotheses could justify the evaluation of some of these treatments for COVID-19 pneumonia, and they are the rationale for some of the trials currently ongoing (Table 2).

However, we are fully conscious of the lack of solid evidence regarding COVID-19 and that every action comes at a price, with potential related complications inflicted by untested treatments. For this reason, at this moment, we discourage the routine use of these treatments, as contradictory experiences and expert opinions have been published [49,50,51,52,53].

## 8. A Look into the Future: COVID-19 as an Infectious Cause of Chronic PH?

Viral infections may lead to the development of PH, with human immunodeficiency virus as a paradigmatic case of this association [6]. Throughout this review, we have described how SARS-Cov-2 causes severe endothelial dysfunctions through mechanisms that remind those implicated in the development of pulmonary vascular remodeling, such as inflammation, hypoxia and microthrombosis [94]. The possibility of a residual endothelial damage leading to the development of chronic pulmonary vascular remodeling in COVID-19 survivors requires special caution and further surveillance. 

Furthermore, the differential procoagulant state in COVID-19 patients may also play a role in the development of PH. Prophylactic anticoagulation effectivity in terms of the reduced incidence of pulmonary embolism in hospitalized patients is low, with a cumulative incidence reaching up to 50% in these patients [29]. These thrombotic events unleashed by the inflammatory response and endothelial dysfunction are prone to an incomplete resolution and could, therefore, lead to the development of CTEPH. A prospective multicentric cohort study is about to be launched in Spain to determine whether these patients are actually at a higher risk to develop CTEPH (PANDEMIC project—Pulmonary hypertension and Embolism in Covid-19, pending for the ethics committee approval).

Last, but not least, the possibility of lung fibrosis leading to the development of severe bronchopathy and group 3 pulmonary hypertension should also be taken in account [95]. However, the long-term prevalence of fibrosis reported in patients infected by SARS-CoV was lower than 10%, and no data describing a high prevalence of fibrosis in Middle East respiratory syndrome patients have been published [96]. 

## 9. Conclusions

Pulmonary damage associated to COVID-19 is a complex merge of different interrelated physiopathological processes in which endothelial dysfunction seems to play a pivotal role, leading to the development of thrombosis and vasomotor disturbances responsible for severe respiratory failure. 

Although the scarcity of available data requires caution, the reported lethality of COVID-19 among PAH and CTEPH patients is low. The epidemiologic and prognostic impacts of COVID-19 in this population should be evaluated through international registries. 

The role of chronic anticoagulation and specific PH therapies as protective measures against the described endothelial dysfunctions requires further investigation with placebo-controlled randomized trials, avoiding futile or even harmful interventions. 

Last, due to severe endothelial dysfunctions, the higher incidences of thrombotic events and possible residual lung fibrosis, COVID-19 patients may require tight follow-ups to discard the possibility of PH as a chronic sequalae of the disease. 

## Figures and Tables

**Table 1:** Most relevant current clinical trials exploring the effectiveness of anticoagulation (source: Clinicaltrials.gov).

Drug	NCT	Intervention	Control
Heparin	04362085 04345848 04406389 04359277 04367831 04377997 04401293 04444700 04360824 04372589 04344756	Therapeutic anticoagulation	Prophylactic anticoagulation
Bivalirudin	04445935	Therapeutic anticoagulation	Standard care (prophylactic heparin)
Rivaroxaban	04416048 04394377	Therapeutic anticoagulation	Standard care (prophylactic heparin)

**Table 2:** The most relevant current clinical trials exploring the effectiveness of specific therapies for pulmonary hypertension in COVID-19 (source: Clinicaltrials.gov).

Drug	NCT	Intervention	Control
Sildenadil	04304313	Sildenafil 100 mg od.	NA
Ambrisentan	04393246	Ambrisentan 5 mg od.	Standard care
Iloprost	04420741	Intravenous iloprost	Saline
	04445246	Inhaled iloprost	Standard care
Nitric oxide	04338828 04388683 04383002 04358588 04290858 04305457 04290871 04337918 04397692 04306393	Inhaled nitric oxide	Standard care
	04421508 04398290 03331445	Inhaled nitric oxide	Pulsed inhaled N_2_
VIP analog	04433546	Oral VIP analog	Placebo
	04360096	Inhaled VIP analog	Placebo
	04311697	Intravenous VIP analog	Placebo
Recombinant ACE2	04375046 04382950	Intravenous ACE2	Standard care
Tocilizumab	04435717 04412291 04346355 04403685 04361552 04322773 04330638 04331808	Tocilizumab	Standard care
	04345445 04377503	Tocilizumab	Corticosteroids
	04412772 04377750 04335071 04372186 04356937 04320615 04335305	Tocilizumab	Placebo
	04332094	Tocilizuman + HCQ + Azytromicin	HCQ + Azytromicin
	04363736	Tocilizumab 4 mg/Kg	Tocilizumab 8 mg/Kg
	04409262	Tocilizumab + Remdesivir	Remdesivir
	04310228	Tocilizumab	Fapiravir

ACE2: Angiotensin-converter enzyme; HCQ: Hydroxicloroquine; NA: Not applicable, od: Once daily.
